# Feeding practices during high-flow nasal cannula: current evidence and clinical implications for respiratory care

**DOI:** 10.3389/fnut.2026.1948830

**Published:** 2026-10-06

**Authors:** Jaber S. Alqahtani, Jood Alkhelb, Alaa Alamri, Leen Alqahtani, Razan Alshehri, Abdullah A. Ghazwani, Hussam M. Almarkhan, Ahmed S. Alqahtani, Meshal H. Alenezi, Abdulelah M. Aldhahir, Rayan A. Siraj, Abdullah A. Alqarni, Hassan Alwafi, Sulaiman M. Almarkhan, Fahad A. Salem, Saad Alrabeeah, Faisal R. Alanazi, Asma O. Alamoudi, Saeed M. Alghamdi

**Affiliations:** 1 Department of Respiratory Care, Prince Sultan Military College of Health Sciences, Dammam, Saudi Arabia; 2 Research and Innovation Institute, Ministry of Defense Health Services, Riyadh, Saudi Arabia; 3 Respiratory Therapy Program, Department of Nursing, College of Nursing and Health Sciences, Jazan University, Jazan, Saudi Arabia; 4 Department of Respiratory Therapy, College of Applied Medical Sciences, King Faisal University, Al-Ahsa, Saudi Arabia; 5 Department of Respiratory Therapy, Faculty of Medical Rehabilitation Sciences, King Abdulaziz University, Jeddah, Saudi Arabia; 6 Faculty of Medicine, Umm Al Qura University, Mecca, Saudi Arabia; 7 King Fahad Military Medical Complex, Ministry of Defense Health Services, Dhahran, Saudi Arabia; 8 Clinical Technology Department, Respiratory Care Program, Faculty of Applied Medical Sciences, Umm Al-Qura University, Makkah, Saudi Arabia

**Keywords:** aspiration, deglutition disorders, enteral nutrition, high-flow nasal cannula, oxygen inhalation therapy, respiratory insufficiency, respiratory failure, feeding

## Abstract

High-flow nasal cannula (HFNC) therapy is increasingly used as a noninvasive respiratory support modality across acute and critical care settings. Although nutritional management is integral to the care of patients with respiratory failure, established guidelines for feeding patients receiving HFNC are lacking, and clinical decisions vary widely, reflecting persistent uncertainty about HFNC’s effects on swallowing function, airway protection, and aspiration risk. This narrative review found that HFNC may alter swallowing biomechanics, particularly at flow rates >40 L/min, potentially increasing the risk of choking and aspiration. Evidence on aspiration risk is heterogeneous, deriving largely from healthy volunteers and pediatric bronchiolitis cohorts. Adult evidence is limited to retrospective studies; the largest found no increase in respiratory deterioration with enteral feeding. Most institutions lack formal feeding protocols, and practice is driven by individual clinician judgment. Areas of consensus include the safety of early enteral feeding in hemodynamically stable patients and the avoidance of routine prolonged withholding of nutrition based solely on HFNC use. Disagreement persists regarding routine swallowing assessment, optimal flow thresholds during feeding, and the prioritization of oral versus tube feeding. Safe feeding in patients receiving HFNC requires a multidisciplinary, individualized approach that balances respiratory support against nutritional adequacy. Prospective multicenter studies are needed to define safe flow thresholds, validate bedside assessment tools, and establish standardized feeding guidelines, particularly in adult and critically ill populations.

## Introduction

High-flow nasal cannula (HFNC) has rapidly become a mainstay of noninvasive respiratory support for adults and children with hypoxemic respiratory failure, including conditions such as COPD, pneumonia, and bronchiolitis (1–3). By delivering a warmed, humidified gas mixture at high flow rates, HFNC improves oxygenation, reduces the work of breathing, and is generally better tolerated than conventional oxygen therapy or other forms of noninvasive respiratory support in many patients (4). One practical advantage often cited by clinicians is that HFNC can potentially allow patients to remain awake, interact, and even eat and drink while receiving substantial respiratory support, which is very different from the experience of intubation or tight-fitting NIV masks (4, 5). In practice, however, decisions about whether and how to feed patients receiving HFNC are less clear-cut than the question suggests.

In theory, preserving oral intake or enteral tube feeding during HFNC should help maintain muscle mass, immune function, and overall recovery, especially in patients with prolonged respiratory failure who are already at high risk of malnutrition (6–8). A recent narrative review notes that many patients receiving noninvasive respiratory support are underfed, reflecting concerns about aspiration, intolerance, or the risk of impending intubation (9). For HFNC specifically, recent physiologic and clinical data suggest that high flow rates may alter upper airway pressures and airflow patterns in ways that can interfere with swallowing and increase the risk of dysphagia in some patients (9). At the same time, pediatric bronchiolitis cohorts have reported low rates of feeding-related adverse events when enteral tube or oral feeds are continued on HFNC, particularly in clinically stable children and at lower flow settings (10). These mixed signals leave bedside teams balancing the benefits of early nutrition against the potential harms of aspiration, desaturation, or clinical deterioration.

Real-world practice reflects this uncertainty. A large survey of clinicians caring for patients on HFNC found that most institutions’ practices are highly variable both between and within centers (11). Despite growing use of HFNC across ICUs, step-down units, and general wards, there are still no clear, evidence-based guidelines for feeding practices in this population (11, 12). As a result, clinicians often rely on local culture, personal experience, and informal thresholds rather than standardized, data-driven guidance.

This narrative literature review aims to synthesize the current evidence on feeding practices in patients receiving HFNC across adult and pediatric populations, various clinical settings, and both oral intake and enteral tube nutrition strategies. Specifically, this review evaluates the available evidence regarding the safety, feasibility, and nutritional adequacy of feeding during HFNC therapy and identifies priorities for future research.

## Methods

This is a narrative literature review. PubMed/MEDLINE, Embase, and Google Scholar were searched for English-language publications from inception to May 2026, using combinations of the terms “high-flow nasal cannula”, “HFNC”, “HFNO”, “oral feeding”, “enteral nutrition”, “swallowing”, “aspiration”, and “dysphagia.” Eligible papers comprised original research, systematic and scoping reviews, as well as clinical practice recommendations relevant to HFNC and feeding practices. Further, reference lists of included articles were hand-searched to identify additional sources. A narrative synthesis was undertaken, and studies were selected for their thematic relevance.

## Physiological effects of HFNC settings relative to feeding

HFNC modifies respiratory physiology in several ways that are directly relevant to feeding safety and tolerance. HFNC delivers heated, humidified gas at flow rates that can approach or exceed a patient’s peak inspiratory demand, generating a low level of positive airway pressure, flushing the nasopharynx, and reducing inspiratory effort. These effects improve oxygenation and reduce the work of breathing, which, in theory, should support safer oral intake by stabilizing ventilation and reducing the need for prolonged fasting or deep sedation. However, the same high flows also alter upper airway mechanics and airflow patterns, with potentially complex consequences for swallowing physiology, airway protection, and gastrointestinal function during feeding (13–17).

From a respiratory standpoint, HFNC provides a modest continuous distending pressure (often 2–6 cmH₂O) that depends on flow, patient size, and mouth position. This positive pressure helps recruit alveoli, increases end-expiratory lung volume, and decreases respiratory rate, thereby unloading the respiratory muscles. Dead-space washout in the nasopharynx further improves ventilatory efficiency, reducing the need for large tidal volumes and allowing a more regular breathing pattern. For patients attempting to eat or receive enteral feeds, these changes may create a more favorable background by reducing dyspnea and improving coordination between breathing and swallowing. Clinically, this is one reason why HFNC is often perceived as more compatible with oral intake than noninvasive ventilation delivered through tight-fitting masks, which can severely disrupt speech and eating (13, 15).

At the same time, several physiological studies in healthy volunteers and patients show that high flow rates can interfere with the fine coordination of swallowing and breathing. In a scoping review of high-flow nasal therapy (14), oral-phase swallowing was selectively disrupted at high flow: modified barium swallow studies (MBSS) impairment profile scores rose significantly only at 60 L/min, while flows of 40 L/min were statistically indistinguishable from baseline (18). The MBSS impairment score is a structured scoring system that quantifies swallowing impairment by rating specific items of the swallow, including oral control, pharyngeal transit, airway protection, residue, and aspiration risk (18). Moreover, penetration–aspiration scores remained stable across all flow rates, indicating that aspiration risk was not directly proportional to flow-induced disruption of the oral phase (14). In a separate volunteer study, Arizono et al. demonstrated a dose-dependent relationship between HFNC flow rate and swallowing difficulty: swallowing frequency declined, and subjective effort increased at flow rates of 20 L/min or higher, with frank choking episodes emerging at 40 L/min or higher (19). In these experiments, higher flows did not consistently increase penetration–aspiration scores, but they did make swallowing feel more effortful and changed respiratory–swallow phase relationships (14). Other studies have found that HFNC can shorten the latency of the swallowing reflex and the time to laryngeal vestibular closure, potentially facilitating earlier initiation of the swallow in response to upper airway stimulation (16). Taken together, these data suggest that HFNC can both facilitate and hinder swallowing, depending on flow rate, individual anatomy, and underlying disease, rather than exerting a uniformly beneficial or harmful effect (Figure 1).

**Figure 1:**
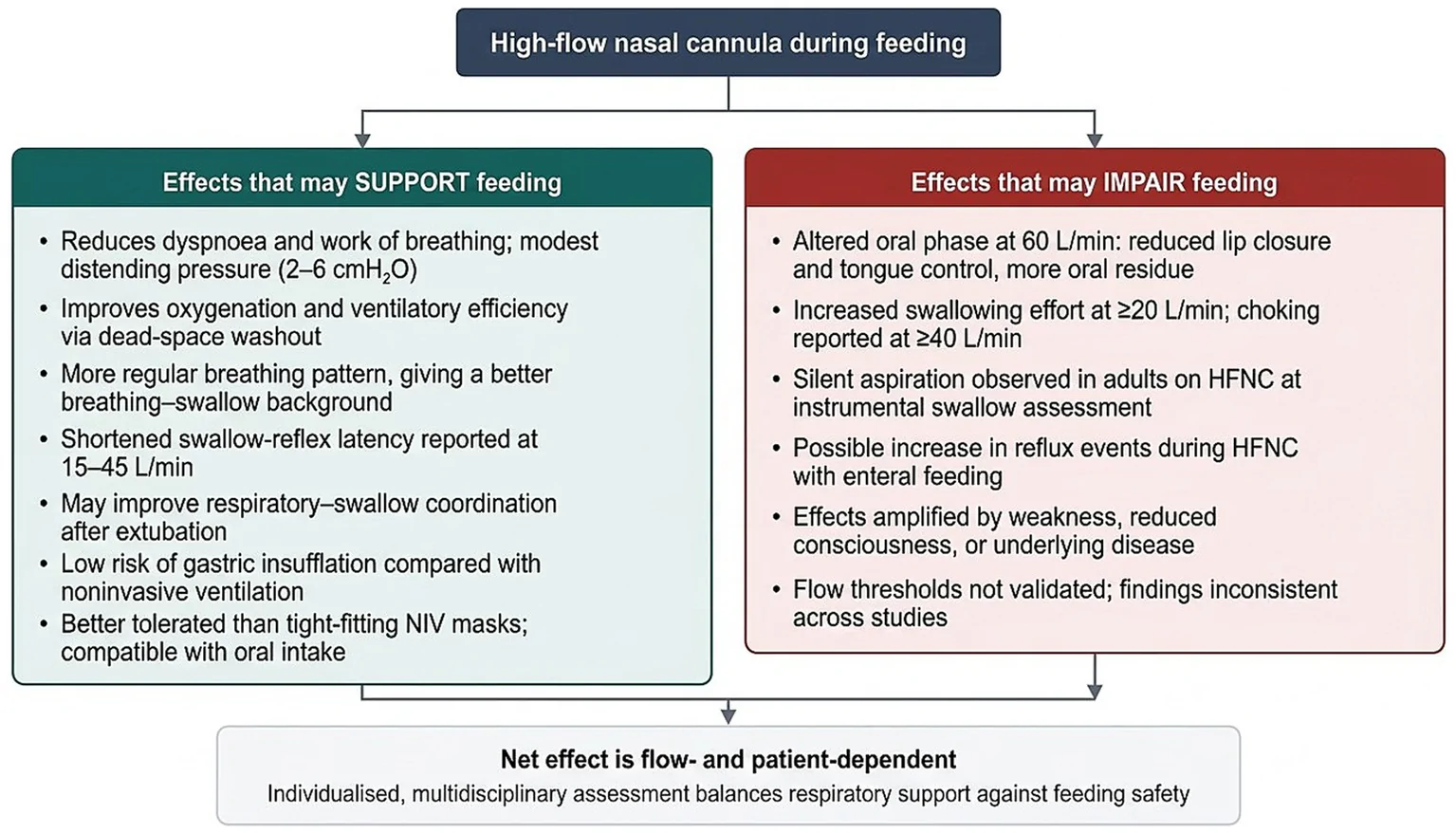
HFNC as a double-edged influence on swallowing and feeding. Physiological effects that may support oral or enteral intake (left) are counterbalanced by effects that may impair swallowing safety (right); the net effect is flow- and patient-dependent, supporting an individualized, multidisciplinary approach. HFNC, high-flow nasal cannula; NIV, noninvasive ventilation.

Preclinical animal models provide additional insight into how HFNC interacts with feeding. In a neonatal lamb model comparing bottle-feeding under nasal CPAP and HFNC, HFNC at 7 L/min did not impair the safety of bottle-feeding or the coordination of sucking, swallowing, and breathing. Although feeding efficiency (feeding duration and milk transfer rate) was actually enhanced under CPAP, HFNC did not worsen these indices compared with spontaneous breathing, and there was no increase in aspiration events (20). These findings support the concept that low-to-moderate HFNC flows may be physiologically compatible with safe oral feeding when respiratory mechanics are otherwise stable. However, translating results from healthy or animal models to acutely ill adults and children with respiratory failure requires caution, as disease-related changes in muscle strength, consciousness, and reflex control may amplify HFNC-related perturbations in swallowing (20).

A key concern when feeding patients on positive pressure or high-flow systems is the risk of gastric insufflation and subsequent aspiration. In spontaneously breathing volunteers, HFNC did not significantly increase gastric volume as measured by MRI, even at high flows, suggesting a low propensity for gastric insufflation in the absence of mask-delivered positive pressure (17). This contrasts with classic noninvasive ventilation, in which higher airway pressures and mask leak are well-recognized contributors to gastric distension and reflux (21). The available physiological data therefore indicate that HFNC’s impact on the stomach is limited, and that feeding-related risks in this context are more likely to be driven by altered swallowing mechanics and respiratory–swallow coordination than by gastric overinflation.

Despite these emerging insights, substantial uncertainty remains about how the physiological effects of HFNC should guide bedside feeding decisions. Existing studies are heterogeneous in design, sample size, flow settings, and outcome measures, and most involve healthy volunteers or small, highly selected patient groups rather than typical critically ill populations (14, 16). Consequently, evidence is insufficient to define safe flow-rate thresholds during oral intake, to identify the conditions under which HFNC may support swallow function, or to establish criteria for preferring enteral tube feeding over oral intake. The effects of HFNC on feeding and swallowing should therefore be interpreted separately by age group, as the relevant clinical considerations differ.

## Safety of oral feeding in patients on HFNC

Evidence to date suggests that oral feeding can be safely maintained in certain patients receiving HFNC, particularly when they are clinically stable and not in significant respiratory distress (22, 23). Concerns about aspiration, swallowing dysfunction, and clinical deterioration have traditionally led many teams to adopt a conservative nil per os approach. Yet the emerging literature, particularly in pediatric bronchiolitis and palliative respiratory failure, indicates a low incidence of serious feeding-related complications when oral intake is permitted under appropriate conditions (22–24). At the same time, the evidence base is largely observational and focused on specific populations, so current conclusions must be applied with careful clinical judgment.

Importantly, the apparent safety of oral intake on HFNC in these studies must be interpreted in the context of careful patient selection and monitoring. Pediatric cohorts generally excluded children with significant comorbidities, neuromuscular disease, or baseline dysphagia, and feeding was initiated once patients were judged clinically stable, with close observation for coughing, gagging, worsening work of breathing, or desaturation during feeds (23, 24). Even in these relatively homogeneous populations, minor adverse events such as transient cough, choking, or increased respiratory effort occurred in a small but non-negligible fraction of children, prompting temporary interruption of feeds in some cases (23). In adults, formal swallow assessments are rarely reported, and changes in oxygenation or dyspnea specifically during meals are often not captured, leaving uncertainties about subclinical aspiration or longer-term pulmonary consequences (11, 22).

Regarding the safety of enteral feeding in patients on HFNC, a single-center retrospective cohort study involving 220 adult patients has been conducted to evaluate this aspect. In this study, 57 patients advanced to noninvasive positive-pressure ventilation, invasive ventilation, or extracorporeal membrane oxygenation. Enteral feeding was associated with a lower likelihood of progression in multivariable analysis (odds ratio 0.33, 95% CI 0.18–0.58), and this association persisted after propensity score matching (odds ratio 0.30, 95% CI 0.15–0.58) (25). Such findings cannot be extrapolated to confirm the safety of oral feeding or the absence of aspiration risk. The link observed in this adult cohort warrants caution given its retrospective, single-center design and potential for unmeasured confounding. This finding generates hypotheses rather than providing clinical recommendations. Instrumental data further temper the picture: in a small retrospective series of adults assessed with MBSS while receiving HFNC, silent aspiration was identified in half of those studied (26), indicating that clinically unremarkable feeding does not exclude aspiration. However, these data were based on a small sample size and therefore require further investigation.

Physiological and mechanistic studies highlight why caution remains warranted despite these encouraging clinical outcomes. Experimental work suggests that higher HFNC flows can alter swallowing biomechanics and respiratory–swallow coordination, raising theoretical concerns about penetration or aspiration at higher flows and in patients with pre-existing dysphagia. However, reported effects are inconsistent across studies (14, 16). Case reports also describe increased reflux events during HFNC and enteral feeding, suggesting that in some individuals, high flow may interact with esophageal function in ways that could increase the risk of aspiration pneumonia (27). These signals have led speech-language pathologists and dysphagia specialists to emphasize individualized assessment rather than a blanket assumption of safety for all HFNC recipients (11).

Because these findings do not resolve into a consistent dose–response relationship, no flow threshold for safe oral intake can currently be derived from the evidence. Figure 2, therefore, sets out a pragmatic framework that stratifies proposed actions by flow rate. It offers a proposed structure for bedside decision-making, rather than as a validated clinical threshold; its cut-points require prospective validation before they can be recommended for routine use. This framework is conceptually derived from limited and retrospective studies and should be interpreted with caution.

**Figure 2:**
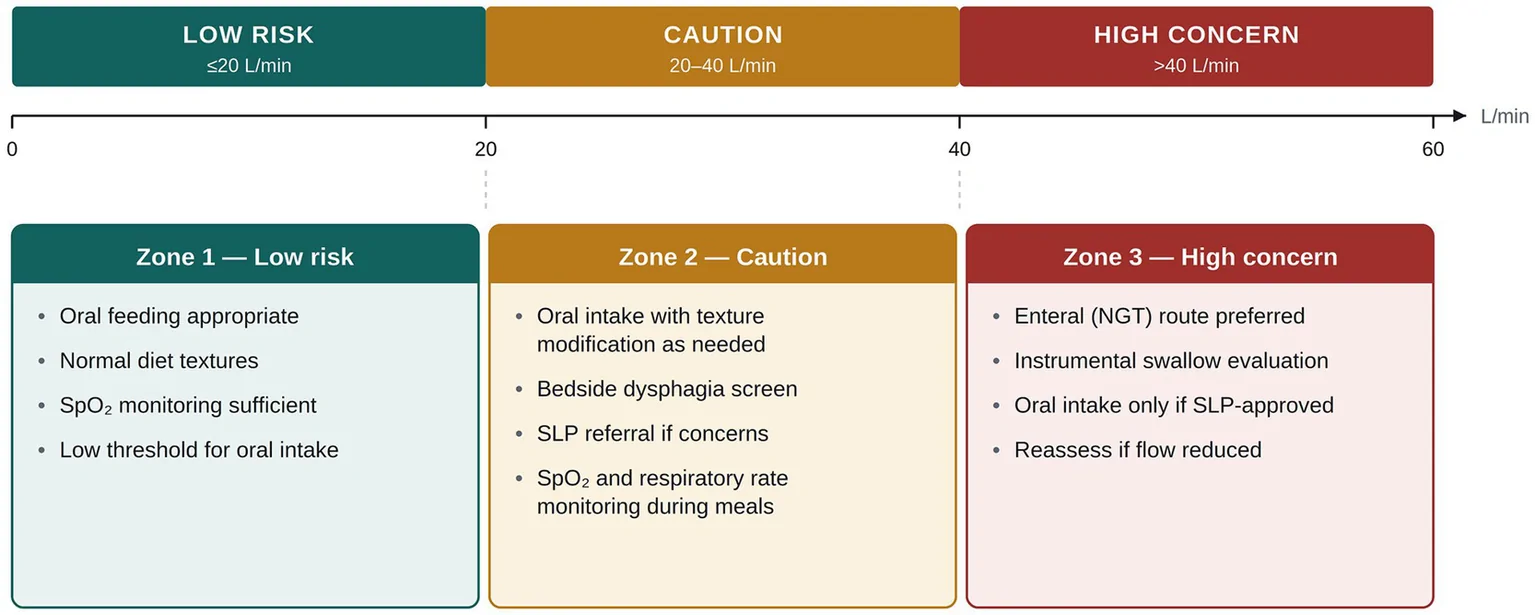
Proposed, evidence-informed framework for stratifying the risk of oral feeding during HFNC therapy according to flow rate. Three zones are defined—low risk (≤20 L/min), caution (20–40 L/min), and high concern (>40 L/min)—each with corresponding feeding-route, swallowing-assessment, and monitoring recommendations. HFNC, high-flow nasal cannula; NGT, nasogastric tube; RR, respiratory rate; SLP, speech-language pathologist; SpO₂, peripheral oxygen saturation.

Overall, the available evidence supports the cautious conclusion that oral feeding is feasible and appears safe in certain patients on HFNC. This applies to infants with bronchiolitis on low-to-moderate flows, to adults in palliative settings, and to enteral feeding for hospitalized adults who are not in significant respiratory distress and are closely monitored during meals. Coughing, choking, or oxygen desaturation during feeding indicates poor tolerance and requires immediate reassessment of the feeding plan. Therefore, feeding decisions for patients receiving HFNC should be individualized, taking into account respiratory stability, swallowing function, and aspiration risk. Altogether, adult evidence remains confined to retrospective, largely single-center cohorts; multi-center studies are absent, objective swallowing evaluations are rarely used, and outcomes beyond progression of respiratory failure, notably aspiration pneumonia and length of stay, remain insufficiently studied. These limitations reinforce the need for a structured synthesis of existing data and for future studies that more rigorously define which patients can safely receive oral nutrition while on HFNC, at what flow thresholds, and under which monitoring protocols.

## Clinical assessment of feeding readiness for patients on HFNC

Clinical assessment of feeding readiness in patients on HFNC should begin with a global appraisal of clinical stability and airway protection. Patients should be hemodynamically stable, without escalating vasopressor or oxygen requirements, and demonstrate a stable or improving respiratory pattern on HFNC, with acceptable work of breathing, respiratory rate, and oxygen saturation at a relatively steady FiO₂ (6, 7, 28). Oral intake should only be considered when the patient is awake or easily arousable, able to follow simple commands, and capable of maintaining airway reflexes, as impaired consciousness and overt respiratory distress are well-recognized risk factors for aspiration in critically ill patients (29, 30). Elderly patients, frailty, presbyphagia, baseline dysphagia, and comorbidities raise the risk of aspiration and undernutrition (6, 31). Accordingly, any patient suspected of having dysphagia should be referred to a speech-language pathologist before initiating oral feeding.

Once basic stability is achieved, structured screening for dysphagia and nutritional risk becomes essential. This is particularly important because post-extubation dysphagia is common: in a prospective single-center cohort of 125 extubated adults, 35% had severe dysphagia on the Gugging Swallowing Screen (32). Bedside swallow screens, such as simple water swallow tests or multi-consistency/volume-viscosity protocols, are recommended as first-line tools to triage which patients may safely begin oral intake. This requires instrumental assessment (videofluoroscopy or fiberoptic endoscopic evaluation of swallowing (FEES)), particularly after prolonged intubation or in those with neurological disease (33, 34). Contemporary ICU nutrition guidelines further advise using structured nutrition-risk tools (e.g., NRS-2002, mNUTRIC) to avoid unnecessary fasting in high-risk patients (6). When oral feeding is inadequate or contraindicated, alternative options such as enteral or parenteral feeding should be considered, and continuous monitoring of oxygen saturation, respiratory rate, and patient comfort is required throughout the feeding session (6, 7, 28). Although HFNC is used across diverse clinical settings, no widely accepted official guidelines for feeding on HFNC currently exist. The lack of standardization leads to inconsistent practices across healthcare facilities, where institutions rely either on local rules or on individual clinical judgment. Figure 3 shows a proposed feeding assessment algorithm for patients on HFNC. However, this proposed algorithm should be interpreted with caution, as it was derived from limited, predominantly retrospective studies. Furthermore, routine clinical assessment should not be treated as a sufficient safety-screening tool because silent aspiration is common and bedside observation alone cannot detect it.

**Figure 3:**
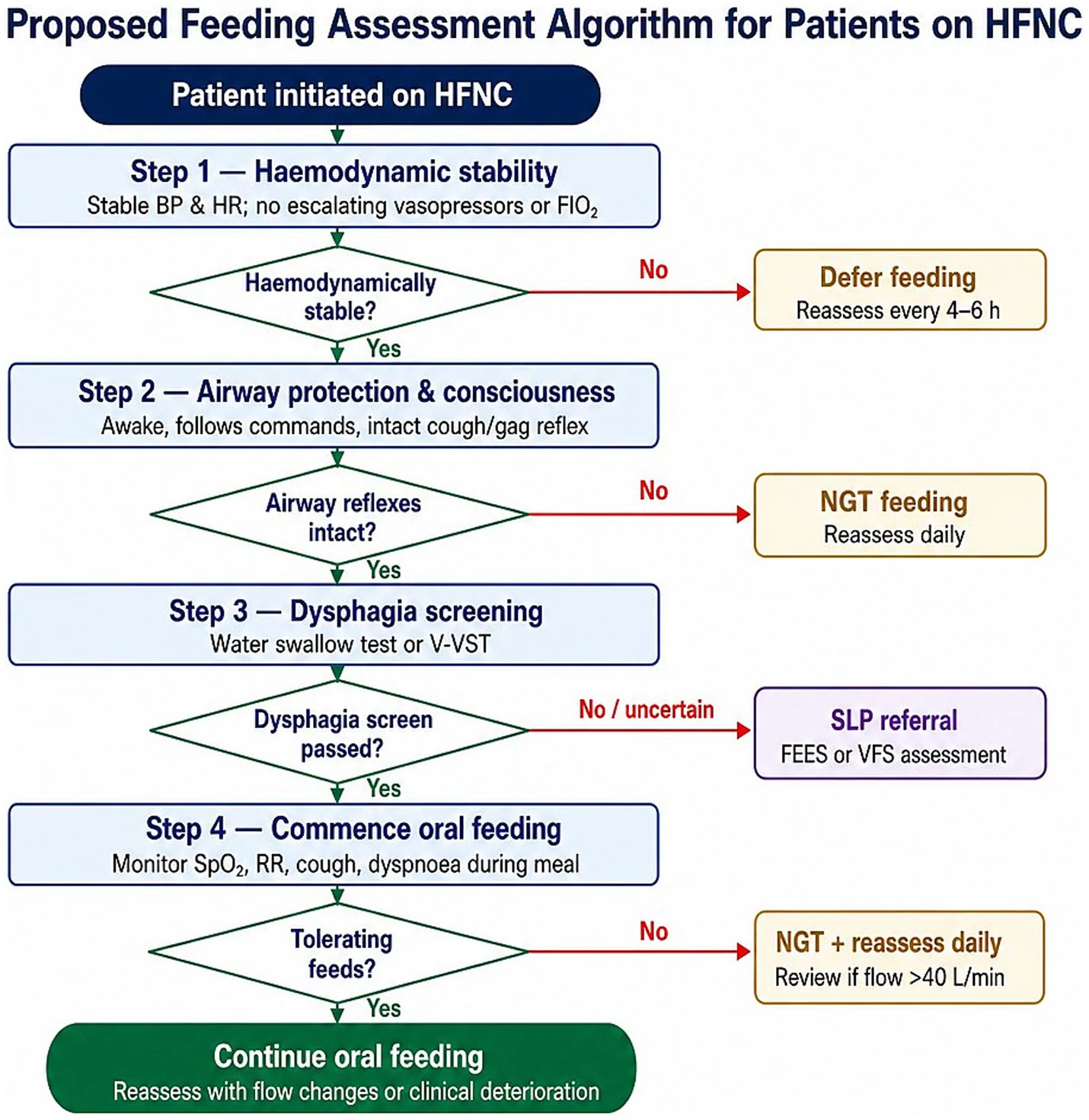
Proposed stepwise clinical pathway for feeding decisions in patients receiving HFNC therapy. Sequential assessment of hemodynamic stability, airway protection and consciousness, dysphagia screening, and feeding tolerance guides progression to oral feeding, with alternative actions (deferral, nasogastric feeding, or speech-language pathology referral) defined at each decision point. FEES, fiber-optic endoscopic evaluation of swallowing; FiO₂, fraction of inspired oxygen; HFNC, high-flow nasal cannula; NGT, nasogastric tube; RR, respiratory rate; SLP, speech-language pathologist; SpO₂, peripheral oxygen saturation; V-VST, volume–viscosity swallow test; VFS, videofluoroscopy.

## Current guidelines, protocols, and variability in practice

Current guidance from respiratory, critical care, and nutrition societies offers only limited specific recommendations on feeding management in patients on HFNC. HFNC is therefore often managed within broader frameworks for noninvasive respiratory support or critical care nutrition. An expert consensus–based clinical practice guideline on nutritional support in critically ill patients recommends oral feeding with careful monitoring of swallowing for critically ill adults receiving HFNC or NIV (35). The recommendation, based on expert opinion, achieved a strong consensus among the panel. The same consensus discourages routine supplemental enteral or parenteral nutrition during the first week of ICU stay, given insufficient evidence (35). The ESPEN guidelines on clinical nutrition in the ICU focus on energy and protein targets for critically ill patients and do not provide HFNC-specific protocols, but they note that patients receiving HFNC may resume oral intake once medically stable (6).

In pediatrics, no guidelines specifically address feeding on HFNC in bronchiolitis or respiratory failure. Observational evidence supports early oral intake or enteral tube feeding in stable patients without an established increased risk of aspiration (36–38). Institutional protocols vary substantially. A large proportion of surveyed centers lack formal protocols and rely on individual clinician judgment for the timing, route (oral vs. nasogastric), and flow adjustments of feeding (11). Pediatric pathways often initiate continuous enteral nutrition (EN) shortly after stabilization on HFNC (e.g., at flows ≤2 L/kg/min) to achieve caloric goals more rapidly (36, 37). Other centers prefer a brief nil per os (NPO) period for observation or allow oral feeds only when respiratory scores are low and stable. Recent work in medically complex infants on prolonged HFNC dependence demonstrated marked heterogeneity even within a single center. Some infants were offered oral feeds with reductions in flow or brief “low-flow sprints,” whereas others remained fully tube-fed because HFNC itself was perceived as a barrier to oral feeding. Only a minority achieved full oral feeds by discharge (39). Variations in practice were evident in a recent survey examining oral feeding practices among healthcare professionals for infants receiving HFNC therapy (40). They reported that inconsistent feeding practices may delay the initiation of oral feeding until respiratory support is removed, even though evidence links delayed feeding to adverse medical and neurodevelopmental outcomes. Furthermore, another survey study reported that it may be appropriate to feed certain clinically stable preterm infants receiving HFNC orally, provided this is done under controlled conditions. Aspiration risk should be determined by patient-specific factors rather than solely by receipt of HFNC (41).

Adult practice varies similarly, ranging from oral intake in stable patients at low intubation risk to tube feeding at higher flows because of dysphagia concerns (11, 26). Current evidence indicates that early enteral or oral feeding may be feasible for carefully selected, clinically stable patients receiving HFNC. The available evidence does not support routine prolonged withholding of nutrition based solely on HFNC use. Although HFNC may affect swallowing mechanics and pharyngeal pressures, these physiological changes do not appear to translate into a clinically significant increase in aspiration risk among suitable patients (25, 42). Disagreement persists about routine instrumental or bedside swallowing assessment. In a multidisciplinary survey, Charlton et al. (11) reported that speech-language pathologists were more than twice as likely as other clinicians to endorse routine pre-feeding bedside swallow assessment (75.5% vs. 32.4%). In particular, 80% of responding institutions lacked formal HFNC feeding guidelines, and substantial uncertainty persisted across several core clinical questions: namely, the optimal flow-rate threshold for safe oral intake, whether HFNC flow should be reduced during feeding, and the relative prioritization of oral versus enteral tube feeding routes (11). The available evidence is largely observational or derived from quality-improvement projects, with a notable absence of high-quality randomized controlled trials (RCTs). The result is persistent practice variation and ongoing concerns about silent aspiration, particularly with prolonged HFNC use or in vulnerable populations (11, 43). Furthermore, multidisciplinary teams including respiratory therapists, nurses, speech-language pathologists, and dietitians play a central role in ensuring safe and effective feeding management for patients receiving HFNC. However, team composition and leadership structures vary across institutions, potentially limiting the consistency and impact of these collaborative efforts (11, 37).

Table 1 summarizes special considerations for feeding patients on HFNC stratified by age. Together, these data indicate that while some local protocols support oral intake and enteral tube feeding on HFNC under defined conditions, there is no consistent, high-quality guideline that integrates HFNC parameters, respiratory stability, and dysphagia risk into unified feeding recommendations, resulting in wide variation in practice between centers and professional groups. All the factors outlined in Table 1 for both pediatric and adult populations should be regarded as suggested guidance derived from existing research rather than validated clinical criteria or official recommendations.

**Table 1:** Age-stratified considerations for feeding in patients receiving HFNC therapy.

Consideration	Pediatric	Shared principles	Adult
Clinical context	Bronchiolitis, viral LRTI (general ward/PICU)	Acute respiratory failure on HFNC	Pneumonia, COPD, ARDS (ICU/step-down)
Flow threshold	≤2 L/kg/min (low risk)	Individualized; reassess at each change	>40 L/min (altered swallowing)
Assessment tool	Clinical stability/respiratory distress score	Bedside swallow screen (water test or V-VST)	V-VST or water swallow test; FEES/VFS if indicated
Preferred feeding route	Oral if stable; NGT if flow >2 L/kg/min	Oral preferred when safely tolerated	Oral if SLP-cleared; NGT if impaired reflexes / high flow
Key swallowing concern	Suck–swallow–breathe coordination	Respiratory–swallow phase coordination	Post-extubation dysphagia; aspiration risk
SLP involvement	Recommended if feeding concerns arise	Strongly recommended for high-risk patients	Routine screen advised if >40 L/min or prior intubation
Evidence base	Moderate; bronchiolitis cohorts predominate	Emerging; largely observational	Limited; few prospective ICU studies
Key guideline	No specific guideline; expert consensus	ESPEN 2019; SRLF/GFRUP 2025	ESPEN 2019; SCCM/ASPEN 2016 (general ICU nutrition)

Pediatric flow thresholds are expressed as L/kg/min to account for body weight; adult thresholds are absolute (L/min). ARDS, acute respiratory distress syndrome; COPD, chronic obstructive pulmonary disease; ESPEN, European Society for Clinical Nutrition and Metabolism; FEES, fiber-optic endoscopic evaluation of swallowing; ICU, intensive care unit; LRTI, lower respiratory tract infection; NGT, nasogastric tube; PICU, pediatric intensive care unit; SLP, speech-language pathologist; V-VST, volume–viscosity swallow test; VFS, videofluoroscopy.

## Barriers and facilitators to safe feeding on HFNC

Feeding practice in patients on HFNC varies widely across healthcare settings, largely because there are no standardized, evidence-based guidelines (11). Decisions on oral intake or enteral tube feeding are therefore made based on individual clinician judgment and local institutional practice rather than from structured protocols (11, 42). The result is variability in clinical decision-making and feeding management. Clinician-related factors, particularly experience with HFNC and an understanding of its respiratory implications, shape feeding decisions (11, 43). Familiarity with HFNC physiology and its potential effects on airway protection can raise clinician confidence in allowing oral intake in clinically stable patients (11). Concerns about aspiration risk and possible respiratory deterioration tend, by contrast, to prompt more conservative approaches that delay oral feeding or initiation of enteral nutrition (23, 42). Institutional factors can also impact feeding practice on HFNC, for instance, access to multidisciplinary inputs, particularly speech-language pathology services, which support structured swallowing assessment (11). Limited access to experienced specialists leads to greater uncertainty and more cautious feeding practices (11, 43); see Table 2.

**Table 2:** Barriers and facilitators to safe feeding in patients receiving HFNC therapy, by level of influence.

Level of influence	Barriers	Facilitators
Patient	Impaired consciousness; reduced airway reflexes; high secretion burden; respiratory distress; neuromuscular disease; baseline dysphagia	Hemodynamic stability; intact cough/gag reflex; alert and cooperative; low FiO₂ requirement; improving respiratory trend; no baseline dysphagia
Clinician	Aspiration-risk concern; limited HFNC physiology knowledge; conservative local culture; inconsistent MDT input; over-reliance on NPO default	Experience with HFNC; MDT collaboration; awareness of early-feeding evidence; structured dysphagia training; SLP involvement
System/Institutional	No formal feeding protocol; limited SLP access; high clinical workload; staffing constraints; no standardized screening tool	Institutional feeding protocol; dedicated SLP service; MDT ward rounds; quality-improvement initiatives; audit-and-feedback culture

Factors were derived from narrative synthesis of the included literature; barriers and facilitators are not mutually exclusive and may co-exist. FiO₂, fraction of inspired oxygen; MDT, multidisciplinary team; NPO, nil per os; SLP, speech-language pathologist.

Patient-related factors anchor feeding-safety decisions (23). Neurological impairment, reduced level of consciousness, fatigue, increased secretion burden, and respiratory distress are associated with increased aspiration risk and impaired swallowing safety (23, 26, 44). Physiological evidence indicates that HFNC may influence the coordination of breathing and swallowing, with downstream effects on swallowing efficiency in vulnerable patients (19, 45).

Operational and environmental factors, such as high workload, staffing limitations, and the complexity of critical care environments, can reduce monitoring capacity during feeding and prompt clinicians to make more conservative feeding decisions (11, 39).

Feeding decisions in patients on HFNC are therefore multifactorial and reflect the interaction of clinical judgment, institutional support, patient condition, and physiological considerations. The case for multidisciplinary collaboration and standardized, evidence-informed feeding protocols is strong: both can improve safety and consistency in practice.

## Gaps in evidence and future directions

Despite the growing clinical use of HFNC, significant gaps remain in the evidence on safe feeding practices with this device. The current literature shows substantial variation in practice and an absence of universally accepted feeding guidelines among various institutions (11). Most available studies are observational or retrospective, which limits causal inference and makes it hard to define safety thresholds. Validated tools to assess feeding readiness in patients on HFNC are lacking, leaving clinicians to rely on subjective judgment (11, 12). Most clinical evidence is drawn from pediatric populations, particularly infants with bronchiolitis (23, 24, 42). The adult literature comprises a small number of retrospective cohorts (25, 26) alongside physiological studies conducted largely in healthy volunteers (14, 16), which limits generalizability to critically ill adults. Physiological evidence indicates that HFNC flow rates may influence swallowing–breathing coordination, but the data come largely from healthy subjects with limited clinical outcomes (14, 19). Important clinical outcomes such as aspiration pneumonia, nutritional adequacy, and hospital length of stay remain insufficiently studied. One included ICU cohort reported that patients on HFNC were significantly underfed, with those maintained on oral intake alone achieving only a fraction of caloric and protein targets, suggesting that permitting oral intake alone does not ensure nutritional adequacy (46).

These gaps point to several priorities for future research. Future research should prioritize prospective, multicenter studies to develop evidence-based guidelines for feeding patients on HFNC. Research priorities include defining flow-rate thresholds for adults with respiratory failure, validating bedside swallow-assessment tools adapted to HFNC, and conducting multicenter randomized trials comparing structured feeding protocols with usual care. Large multicenter cohorts are also needed to confirm the retrospective observation that enteral feeding does not precipitate respiratory deterioration in adults receiving HFNC. Expansion of research into adult and critically ill populations is required to improve external validity and clinical applicability. Finally, future work should incorporate caregiver-reported outcomes and long-term developmental follow-up in infants and children, given emerging concerns that prolonged NPO status or delayed oral experiences on HFNC may contribute to later feeding aversion or delay. Addressing these gaps will be critical to move from opinion-driven, highly variable practice toward consistent, evidence-based protocols for safe and effective feeding in patients supported with HFNC.

## Limitations

Several limitations should be acknowledged. First, this review was limited to English-language research, the included studies were heterogeneous, and the majority of the studies were observational or retrospective, with few RCTs. Second, most clinical evidence on feeding safety during HFNC comes from pediatric populations, particularly infants with bronchiolitis; the existing adult evidence is retrospective and single-center, limiting its generalizability to critically ill adults. Third, several frequently cited mechanistic findings come from healthy volunteer studies rather than from patients with respiratory failure or established dysphagia. Fourth, the search strategy does not provide systematic coverage, which may introduce selection bias and result in missing relevant evidence. Finally, no formal quality appraisal of the included studies was performed.

## Conclusion

Feeding management in patients on HFNC remains complex due to the lack of standardized, evidence-based guidelines. The literature shows considerable variation in clinical practice, shaped by clinician experience, institutional resources, and persistent uncertainty about swallowing safety and aspiration risk. Oral feeding is feasible in carefully selected, clinically stable patients under close monitoring, but decision-making remains partly subjective in the absence of validated assessment tools and standardized criteria. Adult evidence remains confined to retrospective cohorts, limiting the strength of any recommendations for critically ill populations. Safe feeding in patients on HFNC requires a multidisciplinary, individualized approach that balances respiratory support and nutritional adequacy. Future research should develop universally accepted feeding guidelines for patients on HFNC to refine objective assessment tools and define physiological thresholds that support safer, more consistent clinical practice.
